# Supplementary figures and images for: Motor Adaptation Deficits in Children with Developmental Coordination Disorder and/or Reading Disorder
Source: Children (Basel). 2024 Apr 19;11(4):491. doi: 10.3390/children11040491 (PMC11049534; doi:10.3390/children11040491)

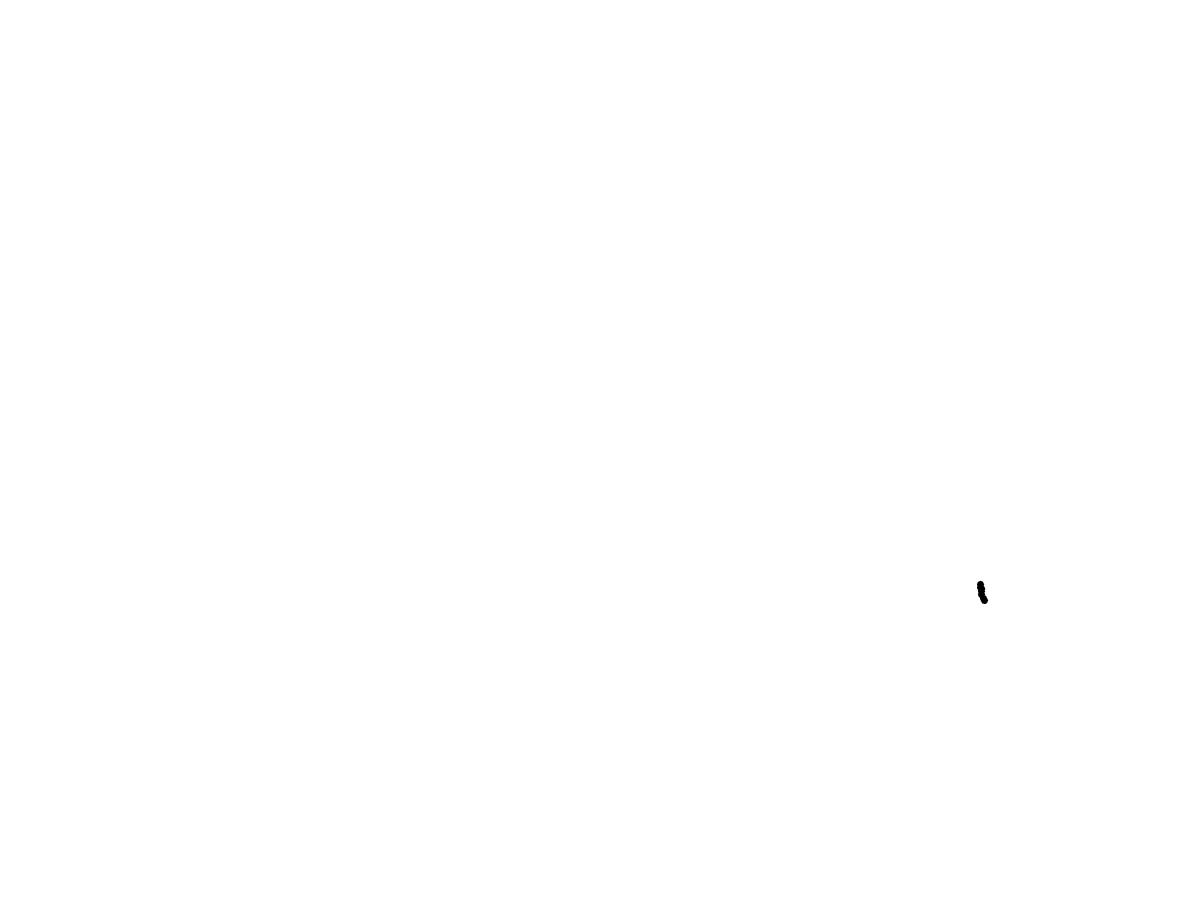

Supplement: Supplementary file 1 [file children-11-00491-s001.zip › SM1_correct_trial.gif]

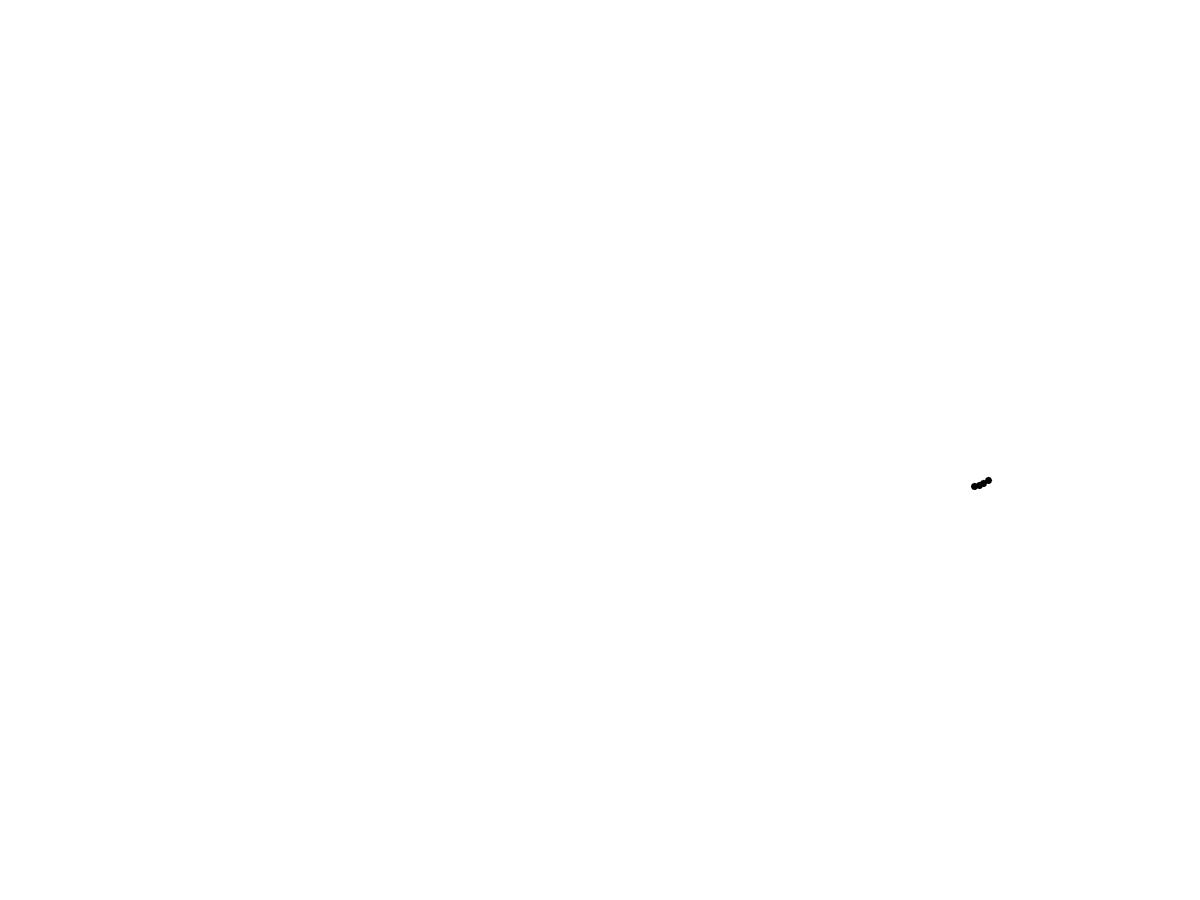

Supplement: Supplementary file 1 [file children-11-00491-s001.zip › SM2_incorrect_trial.gif]
